# Antimicrobial and Other Pharmacological Properties of *Ocimum basilicum*, *Lamiaceae*

**DOI:** 10.3390/molecules29020388

**Published:** 2024-01-12

**Authors:** Kairat Zhakipbekov, Aknur Turgumbayeva, Sholpan Akhelova, Kymbat Bekmuratova, Olga Blinova, Gulnara Utegenova, Klara Shertaeva, Nurlan Sadykov, Kuanysh Tastambek, Akzharkyn Saginbazarova, Kenzhebek Urazgaliyev, Gulbanu Tulegenova, Zere Zhalimova, Zhanylsyn Karasova

**Affiliations:** 1 School of Pharmacy, S.D. Asfendiyarov Kazakh National Medical University, Almaty 050000, Kazakhstan; zhakipbekov.k@kaznmu.kz; 2 Higher School of Medicine, Al-Farabi Kazakh National University, Almaty 050040, Kazakhstan; 3 Department of Pharmaceutical Disciplines, Astana Medical University, Astana 010000, Kazakhstan; ahelova.sh@amu.kz (S.A.); kymbat_0870@mail.ru (K.B.); sadykov.n@amu.kz (N.S.); 4 Department of Organization and Management of Pharmaceutical Business, South Kazakhstan Medical Academy, Shymkent 160001, Kazakhstan; blinova67@mail.ru (O.B.); gulnara64.64@mail.ru (G.U.); klara_shertaeva@mail.ru (K.S.); 5 Department of Biotechnology, M. Auezov South Kazakhstan University, Shymkent 160012, Kazakhstan; tastambeku@gmail.com; 6 Ecology Research Institute, Khoja Akhmet Yassawi International Kazakh-Turkish University, Turkistan 161200, Kazakhstan; 7 Department of Pharmaceutical Disciplines, West Kazakhstan Medical University Named after Marat Ospanov, Aktobe 030012, Kazakhstan; akzharkyn.sab@mail.ru (A.S.); svet.tolga@mail.ru (K.U.); gul_tulegen@mail.ru (G.T.); zere_zhalimova@mail.ru (Z.Z.); karasova_63@mail.ru (Z.K.)

**Keywords:** *Ocimum basilicum*, antimicrobial, antiviral, antioxidant, anti-inflammatory

## Abstract

Since ancient times, various scientists and doctors have utilized different herbs to heal diseases. Due to the rise in drug resistance and the negative effects of chemosynthetic drugs, researchers and the general public around the world have become more interested in medicinal herbs and plant metabolites/extracts. This is due to its non-toxicity and its several health benefits when used to treat diseases in clinical and medical settings. *Ocimum basilicum* is one such plant, possessing a wide range of bioactive phytochemicals including alkaloids, phenolics, flavonoids, tannins, saponins, reducing sugars, cardiac glycosides, steroids and glycosides, as well as complex pharmacological activities, including anti-inflammatory, antifungal, antibacterial, antioxidant, wound healing and antiviral properties. The results of many studies on *Ocimum basilicum* plant extracts are collected and presented in this review. The plant extracts have excellent potential to be used as medicinal raw materials, and exhibit an extensive variety of therapeutic capacities, including antibacterial, antioxidant, wound healing, anti-inflammatory, antifungal, and antiviral properties.

## 1. Introduction

**Figure 1:**
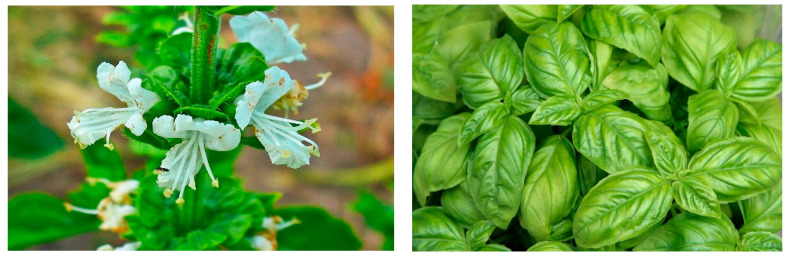
Picture of *Ocimum basilicum*.

**Figure 2:**
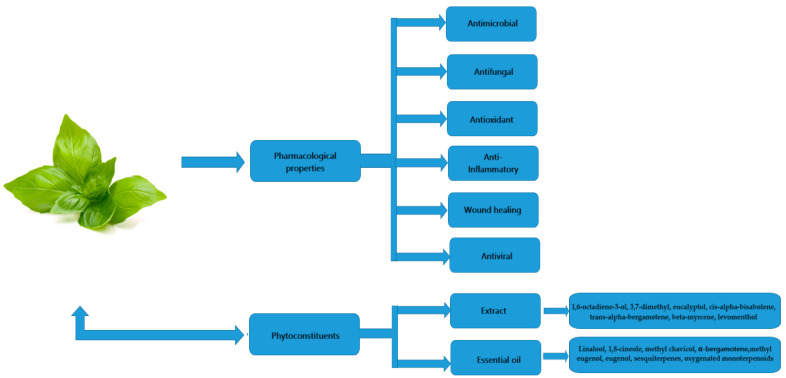
Pharmacological properties and phytochemical components of *Ocimum basilicum*.

Due to increases in drug resistance and the negative effects of chemosynthetic drugs, the interest of researchers and the general public around the world in medicinal plants and plant metabolites/extracts is growing [1,2,3,4]. This is due to the non-toxic nature and numerous health benefits of these substances when used in clinical and medical settings to treat diseases [5,6]. In addition, as a result of scientific progress, many herbs, plant metabolites and phytoconstituents have been developed, promoted and widely used [7,8]. This is due to their recognized and potential health benefits, as well as their medicinal effectiveness against many diseases, both infectious and non-infectious. Interestingly, herbs often show promise in combating antibiotic resistance, helping to prevent dreaded infections [9]. *Ocimum basilicum* is one such example of a whole plant that contains a variety of bioactive phytochemicals that provide various therapeutic effects (Figure 1). *Ocimum basilicum* belongs to the *Lamiaceae* family, previously known as *Labiatae*, which also includes mint (*Mentha* spp.), thyme (*Thymus vulgaris*), sage (*Salvia officinalis*), rosemary (*Salvia rosmarinus*, formerly *Rosmarinus officinalis*) and oregano (*Origanum vulgare*). Terpenoids, flavonoids, alkaloids, tannins, phenolic compounds, cardiac glycosides, saponins, glycosides, reducing sugars and steroids are just a few of *Ocimum basilicum*’s many bioactive metabolites. *Ocimum basilicum* demonstrates various pharmacological activities, including wound-healing, antibacterial, antifungal, antioxidant, and anti-inflammatory qualities (Figure 2) [10,11,12,13]. It is a cheap and widely available medicinal plant, which has been utilized for millennia [14]. This plant grows throughout the world, including in Southeast Asia, India [15], Pakistan, Nepal (Himalayan region) and other countries, as well as tropical, subtropical and temperate regions of Western Asia and Africa [16,17]. This plant is widely available throughout the world, making it easy to purchase and use for many everyday protective purposes [18]. In the systems of Ayurvedic and Unani medicine, it is considered an important element in the treatment of many physiological and lifestyle disorders [19]. In addition, *Ocimum basilicum* is found in many dietary supplements that support and improve health [20,21]. The purpose of this review was to study and familiarize the scientific community with the bioactive compounds and pharmacological properties of *Ocimum basilicum*.

## 2. Methods

We conducted a literature search in scientific engines such as Scopus, Google Scholar, Clarivate, Science Direct, Wiley Online, PubMed, and MDPI to investigate the extracts of *Ocimum basilicum*, as well as its medicinal properties, such as antimicrobial, antiviral, anti-inflammatory, wound healing, antioxidant, and antifungal properties, etc. “*Ocimum basilicum*”, “*Ocimum basilicum* extracts”, “antibacterial characteristics of *Ocimum basilicum*”, “anti-inflammatory qualities of *Ocimum basilicum*”, “antioxidant properties of *Ocimum basilicum*”, “antiviral properties of *Ocimum basilicum*”, and “medicinal uses” were utilized as keywords.

## 3. Phytoconstituents

**Figure 3:**
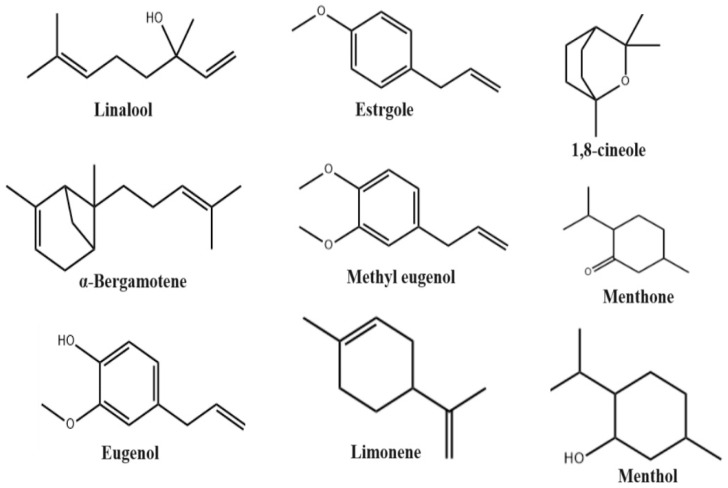
Chemical structures of the main biological active components of *Ocimum basilicum*.

Major studies seeking to determine the phytochemical composition of *Ocimum basilicum* have been conducted using essential oils isolated from *Ocimum basilicum*. *Ocimum basilicum* essential oils are mixtures of aromatic, volatile organic, and natural compounds produced by the plant as secondary metabolites which play a protective role for the plant; they can be extracted from various parts of the plant, including flowers, roots, bark, leaves and seeds [22]. These complex mixtures of volatile secondary metabolites are present as saturated and unsaturated hydrocarbons, ethers, ketones, alkaloids, phenolics, flavonoids, tannins, saponins, reducing sugars, cardiac glycosides, steroids and glycosides [23,24,25,26,27]. However, most studies have shown that the main compounds are estragole, linalool, eugenol, methyl chavicol, methyl eugenol, 1,8-cineole, eucalyptol and bergamotene, at varying concentrations (Figure 3) [28,29,30]. The chemical compounds of *Ocimum basilicum* essential oil undergo quantitative and qualitative changes depending on their genetic characteristics, developmental stage, climatic conditions, drying conditions and storage conditions, as well as the properties of their soils [31]. Table 1 shows the variety of essential oils’ chemical compositions and presents the percentage of each compound present in the essential oil of *Ocimum basilicum*. These variations can be attributed to the area where the oil is collected, the extraction technique, and the portion of the plant from which the oil is extracted.

## 4. Antibacterial Activity

In a study by Adigüzel et al., *Ocimum basilicum* extract’s antimicrobial properties in ethanol, methanol and hexane were tested in vitro. Using disk diffusion and minimum inhibitory concentration (MIC) approaches, 146 microorganisms from 55 distinct bacterial species and four different types of fungus and yeast were investigated. For the 146 bacteria tested, methanol and ethanol extracts demonstrated 10% and 9% inhibition, respectively, while hexane extract showed stronger and more comprehensive antibacterial activity. The ethanol, hexane and methanol extracts’ minimum inhibitory concentrations (MICs) were 125–250 μL/mL, 62.50–250 μL/mL, and 250–500 μL/mL, in this order [43]. In a similar study, the antibacterial activity of *Ocimum basilicum* extracts (ethanol, methanol, and water, respectively) was tested by the presence or absence of obvious growth in the area surrounding the wells. The widest MIC range was 3.125–25 μg/mL for methanol extracts, and then the range gradually narrowed to 6.25–25 μg/mL for ethanol extracts and 12.5–25 μg/mL for aqueous extracts. *B. subtilis* was the organism most sensitive to both of the ethanolic extracts, respectively. The MIC of the methanol extract was 3.125 μg/mL, and the MIC of ethanol extract was 6.25 μg/mL. The MBC results showed that the ethanolic extract had the highest efficacy against *E. coli*, at a concentration of 100 μg/mL, and the aqueous extract had the highest efficacy, at a concentration of 250 μg/mL, against *Staphylococcus aureus* and *E. coli* (Table 2) [44]. Also, among bacterial strains, *Staphylococcus aureus* is highly sensitive to alcoholic extracts with concentrations of 8 mg/mL. Minimal inhibitory doses of 14, 16 and 20 mg/mL of ethanol, methanol, and aqueous extracts inhibited vancomycin-resistant enterococci [45]. Moreover, the flower oil extract showed the strongest antibacterial properties against *Staphylococcus aureus*, with the highest inhibition zone, of 15.47 mm, the lowest minimum inhibitory concentration MIC (0.09 μg/mL) and a correspondingly low minimum bactericidal concentration MBC (0.19 μg/mL) [46]. *Ocimum basilicum* extract has the ability to inhibit bacterial growth due to its rich content of phenolic compounds, such as eugenol, methyl eugenol, benzoic acid, 4-hydroxybenzoic acid, salicylic acid and phenol. Phenolic compounds have many effects on microorganisms, such as altering the permeability of microbial cell membranes due to the accumulation of hydrophobic groups in the phospholipid bilayer, disrupting membrane integrity, causing leakage in intracellular components, and ultimately leading to cell death. Phenolic compounds can also bind to enzymes and inhibit their functions, including those related to protein, DNA, and RNA synthesis. *Ocimum basilicum* extract also contains terpene compounds such as phytol; 3,7,11,15-tetramethyl-2-hexadecen-1-ol; lupeol; and beta-amyrin, which act on and destroy the cell membranes of microorganisms. Furthermore, antibacterial activity is associated with the presence of fatty acids. *Ocimum basilicum* extract is rich in saturated and unsaturated fatty acids with long carbon chains of 16 or more. Fatty acids with carbon chains of six or less inhibit Gram-negative bacteria, whereas Gram-positive bacteria are inhibited by fatty acids with carbon chains greater than twelve, and yeasts are inhibited by fatty acids with carbon chains of ten to twelve.

*Ocimum basilicum* essential oil had strong antibacterial effects against all strains of Gram-positive and Gram-negative bacteria tested in a study of the oil’s effect on the growth of eleven different types of microorganisms. Compared with the commercial antibiotic ciprofloxacin, *Ocimum basilicum* essential oil showed greater antibacterial activity against *S. enterica*, *P. stuartii*, coagulase-positive *Staphylococci*, and group *D streptococci*. Additionally, compared with the commercial antibiotic gentamicin, *Ocimum basilicum* essential oil showed greater antibacterial activity against species of *Salmonella*, *E. coli*, *S. enterica*, *P. stuartii*, coagulase-positive *Staphylococci and* group *D streptococci*. The smallest inhibition zone was 9.66 mm, for *B. cereus*, and the largest was 40.00 mm, for coagulase-positive *Staphylococcus*, which indicates a strong inhibitory effect of *Ocimum basilicum* essential oil [47]. On the other hand, the most sensitive microorganisms were *B. subtilis* and *S. aureus*, with the biggest inhibition zones (22.2–24.4 mm) and the lowest MIC values (0.9 mg/mL) (0.8 mg/mL), respectively, against nine “pathominimum” inhibitory concentrations (MICs). These findings are based on the antimicrobial activity levels of essential oils obtained from *Ocimum basilicum*, which was collected seasonally in winter and autumn. Less activity was noted against *M. mucedo* with the smallest zones of inhibition (9.7–13.6 mm) and the highest MIC values (3.8–5.1 mg/mL). At MIC values of 0.3–1.9 mg/mL, linalool isolated from seasonally collected essential oils of *Ocimum basilicum* exhibited greater antibacterial activity than the whole oils. Research efforts have indicated that linalool and *Ocimum basilicum* essential oil are more effective against bacterial strains than antifungal strains. Linalool and essential oils often showed greater antibacterial activity against Gram-positive microorganisms [24]. It was also found that the microorganisms *S. aureus*, *B. subtilis*, *A. fumigatus*, *S. faecalis*, *S. epidermidis*, *P. chrysogenum* and *A. niger* were more sensitive to the essential oil, with MBC values of 0.143 ± 0.031, 0.260 ± 0.080, 0.312 ± 0.171, 0.364 ± 0.127, 0.416 ± 0.415, 0.416 ± 0.161 and 0.442 ± 0.207 mg/mL, respectively. At the same time, the microorganisms *M. flavus*, *M. luteus*, *P. mirabilis*, *P. vulgaris* and *P. aeruginosa* turned out to be moderately sensitive, with MBC values of 0.520 ± 0.161, 0.572 ± 0.127, 0.781 ± 0.382, 0.833 ± 0.322 and 0.937 ± 0.342, respectively. Microorganisms *E. aerogenes*, *S. marcescens*, *S. typhimurium*, *E. coli* and *K. pneumoniae* were less sensitive and had higher MBC values (MBC > 1.0 mg/mL). Essential oils tested for their antibacterial properties showed greater effects against Gram-positive bacteria [48,49]. According to another study, among Gram-positive bacteria, *Bacillus subtilis* exhibits maximum antimicrobial activity at a concentration of 25 μL of pure oil (without dilution), with a zone of inhibition diameter of 41.50 ± 0.31 mm, whereas diluting the essential oil 1:1 and 1:5 also showed good results: 39.00 ± 0.53 mm and 33.00 ± 0.26 mm, respectively. For *Enterococcus faecalis* in pure oil/1:1/1:5 dilution, the inhibition zone diameter (IZD) was 38.00 ± 0.24 mm, 31.66 ± 1.06 mm and 28.00 ± 0.53 mm, respectively. The *Staphylococcus aureus* IZD was 34.00 ± 0.31 mm for discs soaked in 25 μL of pure essential oil and decreased with increasing essential-oil dilution. In the present study, the antimicrobial activity of *Ocimum basilicum* essential oil was also evaluated against three Gram-negative bacteria, including *Salmonella typhimurium*, *Klebsiella pneumoniae* and *Escherichia coli*. However, unlike other studies, all Gram-negative bacteria were susceptible to the essential oil of *Ocimum basilicum*. Among the bacteria studied, *S. typhimurium* had the largest inhibitory zone diameter, of 33.00 ± 1.06 at pure oil concentration, followed by 27.00 ± 1.41 mm and 22.25 ± 1.77 mm at ether dilutions of the oil of 1:1 and 1:5 respectively. *K. pneumoniae* and *E. coli* showed IZDs of 31.50 ± 0.70 mm and 30.00 ± 0.35 mm, respectively, at the above-mentioned net essential-oil concentrations [50,51].

Most essential oils tested for antibacterial properties also demonstrated higher efficacy against Gram-positive bacteria in comparison to Gram-negative bacteria. This is because the presence of an outer membrane composed of lipopolysaccharides allows Gram-negative bacteria to protect themselves by limiting the penetration of hydrophobic compounds such as essential oil. Thus, the essential oil may be unable to properly attack the phospholipid layers of bacterial cells, compromising their permeability and integrity. *Ocimum basilicum* essential oil showed high antibacterial activity against Gram-positive bacteria, which is due to the essential oil’s main components, namely, phenolic component—estragole and monoterpenoid compound—linalool. The presence of these components in essential oil may promote antimicrobial activity by disrupting the permeability and integrity of bacterial membranes, producing intracellular ATP and potassium ion leakage, and leading to cell death.

Studying the antimicrobial potential of *Ocimum basilicum* leaf essential oil (OEFOb), its main compound estragole (ES) and its estragole/β-cyclodextrin complex (ES/β-CD) in adult zebrafish (aZF) showed good results. Antimicrobial activity was assessed by the broth microdilution method, with determination of the minimum inhibitory concentration (MIC) and evaluation of the potentiation effect in vitro adapted from the in vivo infection method for *S. aureus* and *E. coli* in aZF. According to the results, OEFOb showed MIC values of 2048 μg/mL against the strains in the in vitro assay; on the other hand, estragole and its ES/β-CD complex showed MIC values of 1024 μg/mL. An in vivo infection model of *S. aureus* and *E. coli* on aZF was established at 24 and 48 h post-challenge with bacterial inoculum. Oral administration of the OEFOb, ES, and ES/β-CD complex did not cause mortality in aZF during up to 48 h of testing, and also reduced infections induced by *S. aureus* ATCC 25923 and *E. coli* ATCC 2592 in aZF, demonstrating clinically significant effects. When testing the synergistic effects, the complex of OEFOb, ES, and ES/β-CD was significantly effective against the strains studied, with the ES/β-CD complex giving more significant results when combined with gentamicin [52].

## 5. Antifungal Activity

The antifungal activity of the ethanolic extract of *Ocimum basilicum* was tested using the agar plate method against *F. verticillioides*, *F. subglutinans*, *F. proliferatum* and *Fusarium oxysporum* isolated from the pomace. The growth of *F. subglutinans* (44.30 and 33.37%, respectively) and *F. proliferatum* (29.27 and 24.74%, respectively) was significantly inhibited by extract concentrations of 0.35 and 0.70% (*v*/*v*), while other species were less sensitive. *Ocimum basilicum* extract at a concentration of 1.50% (*v*/*v*) completely inhibited the growth of the *Fusarium* species tested. All species tested showed a reduction in aerial mycelial growth at higher concentrations (0.35 and 0.70% (*v*/*v*)). *F. proliferatum* and *F. verticillioides* had strong medium pigmentation. Microscopic examination of the samples showed hyphal deformations as well as frequent signs of fragmentation, thickening and decreased sporulation [53]. The crude methanol fraction of *Ocimum basilicum* was effective against eight different fungal strains. Even at the lowest dose (1 mg/mL) of the extract used, mild to moderate growth inhibition (10% to 65%) was observed. Since only 10% growth inhibition was observed at this dosage, *Candida albicans* appeared to be more resistant. For *Curvularia lunata* species, moderate inhibition (27%) was observed at a dose of 1 mg/mL. At a dose of 1 mg/mL, the effect of *Penicillium* was sharply inhibited (65%). Suppression of mycelial development was observed for all strains except *Curvilaria lunata* (43%) and *Candida albicans* (17%) at doses of 3 mg/mL [54]. Antifungal activity of aqueous extracts of *Ocimum basilicum* showed that a concentration of 10 mg/mL could inhibit the growth of *Fusarium oxysporum*, a fungus known to cause wilting in crops. The antifungal properties of *Ocimum basilicum* extract are attributed to the presence of tannins, known antimicrobial agents that can inhibit the growth of microorganisms by precipitating microbial protein and depriving them of nutrients necessary for their growth and development. Tannins are acrid, bitter plant polyphenols that either bind and precipitate or compress proteins [55].

*Ocimum basilicum* (Thai basil) has been tested against seven different species of rice pathogenic fungi, including *Alternaria brassicicola*, *Bipolaris oryzae*, *Aspergillus flavus*, *Fusarium moniliforme*, *Pyricleriarisea*, *Fusarium proliferatum* and *Rhizoctonia solani*, and it has been determined that essential oil of *Ocimum basilicum* inhibits spore germination and mycelial growth. The experiment was performed in vitro using PDA (potato dextrose agar) and CRD (complete randomized design) in triplicate. *Ocimum basilicum* oil at a concentration of 0.6% *v*/*v* showed the greatest inhibition of mycelial growth of *F. moniliform* (100%), *F. proliferratum* (49.6%) and *P. grisea* (100%), as measured by mycelial growth inhibition. According to data obtained 7 days after inoculation at 25 ± 2 °C, *A. flavus*, *A. brassicicola* and *B. oryzae* were inhibited by 59.25%, 94.62 and 97.40% at 2.0% *v*/*v*, respectively. However, *Ocimum basilicum* essential oil was ineffective against *R. solani*. *Ocimum basilicum* essential oil showed effectiveness against *F. monoliform* (91.31%) and *A. brassicicola* (99.74%) at a concentration of 0.8% *v*/*v*, according to the inhibition of spore germination recorded at a temperature of 25 ± 2 °C 24 h after inoculation [56].

The antifungal and antibacterial mechanisms of action of essential oils are comparable. The antifungal activity of *Ocimum basilicum* essential oil is mainly due to its main compounds. Numerous studies have shown that certain components of essential-oil mixtures, such as eugenol, linalool, and methyl chavicol, act synergistically in some cases, and, in others, functioning as the leading components of the mixture, damage cell membranes and affect a wide range of other cellular functions, such as energy synthesis. Proton-pump disruption, drop in membrane potential, and ATP depletion are associated with the antifungal effects of the above-mentioned key compounds of *Ocimum basilicum*. In addition, the effects of the activity of *Ocimum basilicum* essential-oil compounds are coagulation of cellular contents, leakage of cytoplasm, and, ultimately, apoptosis or cell necrosis leading to cell death [57].

## 6. Antioxidant Activity

The total antioxidant activity levels of aqueous (WEB) and ethanolic (EEB) extracts of *Ocimum basilicum* were comparable to those of reference compounds such as BHA, BHT, α-tocopherol, and Trolox, which was determined by the iron thiocyanate method, and the activity levels increased steadily with increasing concentrations. WEB, EEB, and reference compounds showed effective antioxidant activity. The percentages of inhibition for these samples in the linoleic acid system were 94.8%, 97.5%, 97.1% and 98.5%, respectively, and were higher than that of α-tocopherol (70.4%) at the same concentration. The percentages of inhibition of peroxidation in the linoleic acid system by concentrations of WEB and EEB of 25 and 50 μg/mL were 73.8%, 94.8%, 90.8% and 95.0%, respectively. At the same time, the inhibition percentages of 50 μg/mL concentrations of BHT, BHA, and α-tocopherol were 97.1%, 98.5% and 70.4%, respectively. The free-radical scavenging activity of these samples also grew as concentration levels rose. Inhibition of the formation of superoxide radicals by WEB and EEB is statistically similar to that of the reference compounds (*p* > 0.05). The percentages of inhibition of superoxide production by WEB and EEB concentrations of 50 μg/mL were 97.9% and 95.4%. At the same concentration, BHA, BHT, and α-tocopherol exhibited superoxide radical scavenging activity levels of 69.6%, 82.2% and 75.4%, respectively. The superoxide radical scavenging activity of these samples was in the following order: WEB > EEB > BHT > α-tocopherol > BHA [58]. In a similar study, ethanol extracts of *Ocimum basilicum* leaves showed exceptional antioxidant activity in DPPH (82.4%), H_2_O_2_ (54.0%) and FRAP (237 µmol Fe/g) tests. Additionally, extracts prepared in water and dichloromethane showed moderate levels of antioxidant activity, while extracts prepared in n-hexane showed negligible results in all three assays, namely, DPPH (32.4%), H_2_O_2_ (12%) and FRAP (316 µmol Fe/g). Based on the results of antioxidant studies, the authors concluded that the higher levels of radical activity of ethanolic extracts of *Ocimum basilicum* leaves may be due to the presence of phenolic compounds, flavonoids and tannins [59].

*Ocimum basilicum* extract has the ability to protect LDL from oxidation. By reducing cholesterol synthesis and regulating the activity of surface scavenger receptors, ethanolic extract of *Ocimum basilicum* has the ability to reduce foam cell formation [60]. The antioxidant effect of *Ocimum basilicum* may provide ovarian protection against reactive oxygen spaces (ROS), and is useful in protecting tissues and reducing the carcinogenic effects of electromagnetic fields. *Ocimum basilicum* extract significantly reduced the number of apoptotic granulosa cells, whereas exposure to electromagnetic fields (EMF) at 50 Hz caused a significant increase in the percentage of apoptotic granulosa cells. Thus, *Ocimum basilicum* extract can be considered as an antioxidant therapy against EMF exposure in industrial areas [61]. Notable inhibition of lipid peroxidation (LPx) in liposomes and potent scavenging activity were shown by various *Ocimum basilicum* extracts, including H_2_O, EtOAc, and n-BuOH extracts. In addition, CHCl_3_ and Et_2_O extracts showed better neutralization of 2,2-diphenyl-1-picrylhydrazyl (DPPH), hydroxyl (OH), nitric oxide (NO), hydrogen peroxide radicals (H_2_O_2_) and superoxide anion (O_2_•-) (weak influence). The weak pro-oxidant properties of the plant are indicated by the effect of extracts on the generation of OH radicals and inhibition of LPx [62]. Hepatorenal toxicity caused by acetaminophen may be reduced by *Ocimum basilicum* extract [63], which is due to the antioxidant properties of the extract and may be associated with inhibition of lipid synthesis in the liver [64].

Essential oils of *Ocimum basilicum* from different places in Egypt showed good radical scavenging activity levels, although lower than that of the synthetic antioxidant BHT (IC_50_ = 6.80 mg/mL). The highest level of radical scavenging activity was recorded for the essential oil from the Minia region, with an IC_50_ value of 11.23 mg/mL, followed by 17.52 and 55.15 mg/mL, for the essential oils from the Assiut and BeniSuef regions, respectively. These differences were due to differences in the environmental conditions. In addition, a low correlation (R2 = 0.441) was found between the antioxidant activity data obtained from DPPH analysis (IC_50_) and the total phenolic content levels of *Ocimum basilicum* essential oils obtained from different locations. It is believed that the increase in radical activity demonstrated by the Minia region essential oil is associated with its high content of eugenol, the concentration of which is approximately twice and three times that found in essential oils from the Assiut and BeniSuef regions, respectively [65].

*Ocimum basilicum* essential oils were shown, in a study by Hussain et al., to be able to convert the stable violet-colored DPPH radical to yellow-colored DPPH-H. Essential oils of *Ocimum basilicum* collected in the winter and spring crops exhibited higher radical scavenging activity levels than those found in oils collected in the autumn and summer, showing IC_50_ values of 6.0, 6.7, 4.8 and 5.3 lg/mL, respectively. Also in this study, linalool, the primary constituent of *Ocimum basilicum* essential oil, examined in a same setting, showed lower antioxidant activity (IC_50_ = 16.4 μg/mL) than the whole oil. The DPPH scavenging activity of winter and spring samples of *Ocimum basilicum* essential oil showed levels comparable to that of the synthetic antioxidant BHT. In addition, *Ocimum basilicum* essential oils prevented linoleic acid from oxidizing, by 80.3–91.2%. The effectiveness levels of essential oils of winter (91.2%) and spring (90.3%) crops were comparable to the effectiveness of BHT (91.1%). At the same time, the values observed for the summer (80.3%) and autumn (84.3%) samples were significantly (*p* < 0.05) lower than for the BHT. Linalool also showed 75.2% inhibition of peroxidation, which demonstrated comparatively less activity than the whole oil. The entire essential oil exhibited higher antioxidant activity than did the separate components, indicating a synergistic interaction between essential-oil components [66]. A similar study conducted by Shafique et al. confirmed these results. The essential oil of *Ocimum basilicum* reduced the stable 2,2-diphenyl-1-picrylhydrazyl radical (DPPH) to yellow DPPH-H, with an overall DPPH removal effect of 96.16% at 100% concentration, as opposed to 85.60% with BHT. Furthermore, *Ocimum basilicum* essential oil demonstrated greater antioxidant activity than did BHT at all concentration levels, ranging from 20 to 100%. At 100% concentration, essential oils had 12.33% more antioxidant activity than did BHT. It was found that the essential oil’s ability to scavenge DPPH radicals increased significantly as essential-oil concentrations rose, indicating that the essential oil had a higher capacity to donate hydrogen [67].

The antioxidant capacity of volatile aglycones and essential oil of *Ocimum basilicum* was compared with that of the known synthetic antioxidant BHT and pure eugenol, in a study conducted by Politeo et.al. Eugenol had the best radical scavenging ability (EC_50_ = 0.096 g/L). *Ocimum basilicum* essential oil and the well-known synthetic antioxidant BHT showed similar capacities (EC_50_ = 1.378 g/L and 0.908 g/L), while the capacity of *Ocimum basilicum* volatile aglycones was significantly lower (EC_50_ = 3.338 g/L). The radical scavenging capacity of the corresponding mass of eugenol in the essential oil was comparable to that of pure eugenol (EC_50_ = 0.099 g/L), while the EC_50_ for the corresponding mass of eugenol in the volatile eugenol fraction was 1.950 g/L. The reducing power of volatile aglycones was also comparable to, but less than, the reducing power of essential oil and BHT [24].

The best anti-inflammatory and antioxidant potential was demonstrated by the *O. basilicum*/*O. gratissimum* essential-oil combination. Gratissimum occurs by inhibiting all cyclooxygenase isoforms by inhibition of ≥98% of cyclooxygenase 1 and ≥67% of cyclooxygenase 2 [48]. Essential oils from *Ocimum basilicum* growing in Iraq showed good antioxidant effects. In an antioxidant study, the essential oil demonstrated strong inhibition of autoxidation by linoleic acid, by 110.8%, while scavenging of the DPPH radical yielded an IC_50_ value of 145.35 μg/mL [68].

The phenolic content of *Ocimum basilicum* essential oils directly contributes to their antioxidant effects. Because of their hydroxyl groups, phenolic compounds have the capacity to scavenge free radicals, making them extremely significant components. The radical activity of the essential oil is associated with the content of eugenol and methyl chavicol, the main representatives of phenolic compounds, in the essential oil of *Ocimum basilicum*. Additional methoxy groups significantly increase antioxidant activity, which explains the high antioxidant activity of eugenol. It has been established that the decrease in eugenol content in the essential oil of *Ocimum basilicum* leads to a decrease in its antioxidant capacity. Methyl chavicol exhibits a more moderate antioxidant activity than does eugenol. The antioxidant activity of *Ocimum basilicum* essential oils is not only due to phenolic components. It is also due to the presence of other, secondary, antioxidant metabolites. Linalool, one of the main terpenoid compounds of *Ocimum basilicum* essential oil, has antioxidant activity comparable to that of the synthetic antioxidant BHT. However, the essential oil of *Ocimum basilicum*, containing methyl chavicol (45.8%) and linalool (24.2%) as the main components, exhibited very strong radical scavenging activity, reducing the formation of DPPH radicals. Thus, the main component does not always determine the antioxidant activity of an essential oil. Components present in lower concentrations may also act synergistically with other active compounds to enhance antioxidant activity [69,70].

## 7. Anti-Inflammatory Activity

A comparison of the anti-inflammatory activity levels of the ethanol extract of *Ocimum basilicum* and diclofenac sodium showed a significant reduction in the lysis of HRBC under hypotonic conditions by the ethanol extract, compared with diclofenac sodium, over the entire range of concentrations tested. The trend towards reduced hemolysis was directly dependent on concentration: higher concentrations of *Ocimum basilicum* ethanol extract and diclofenac sodium resulted in better reduction of HRBC lysis. At a diclofenac sodium concentration of 50 μg/mL, hemolysis was 64.08 ± 1.33%, and further decreased to 39.81 ± 2.95% at a concentration of 1000 μg/mL. However, the ethanolic extract of *Ocimum basilicum* showed 27.18 ± 2.98% lysis of HRBC at 50 μg/mL and 1.94 ± 1.43% hemolysis at 1000 μg/mL. At a concentration of 50 μg/mL, for an ethanolic extract of *Ocimum basilicum*, protection against hemolysis was 2.03 times higher, compared to diclofenac sodium. At concentrations of both compounds of 1000 μg/mL, the HRBC protective potential of *Ocimum basilicum* extract was 1.63 times higher, compared to that of diclofenac sodium. The ethanol extract demonstrated a significant membrane-stabilizing effect by inhibiting hypotonicity-induced erythrocyte membrane lysis. Lysosomal membrane stabilization is an important step for preventing the process of inflammation. The anti-inflammatory effect has been achieved due to the phytochemicals present in *Ocimum basilicum*, such as flavonoids, tannins, etc., which, either separately or in combination, improve the anti-inflammatory action overall. Phenolic compounds are known to inhibit the synthesis of a pro-inflammatory compound during inflammation. Flavonoids block various molecules that promote inflammation, such as COX, cytokines, nuclear factor B and matrix metalloproteinases. Tannin, especially condensed tannin (proanthocyanidins), has beneficial effects on humans because it neutralizes ROS and free radicals present in the living system [71]. Other studies have evaluated the anti-inflammatory activity of an aqueous extract of *Ocimum basilicum* (OBW) in human macrophage (RAW264.7) and chondrosarcoma (SW1353) cell lines, as well as in primary human chondrocytes, to determine efficacy in the treatment of osteoarthritis (OA). In RAW264.7, OBW was effective in reducing prostaglandin (PGE2) (70.8% ± 0.93) and nitric oxide (NO) (35% ± 0.22) production. Levels of iNOS (inducible nitric oxide synthase) protein expression decreased 71.4 ± 2.43%, in tandem with the decrease in NO. OBW significantly decreased nuclear factor kappa B (NFκB) (79.28% ± 1.8) and cyclooxygenase (COX)-2 (83.87% ± 0.95) protein levels. Similarly, OBW significantly reduced the production of PGE2 (76.11% ± 5.5) and leukotriene (LTB4) (59.6% ± 0.22) in SW1353 and chondrocytes. In chondrocytes, OBW decreased the production of matrix metalloproteinases (MMPs) -2 (58.49% ± 1.41), -9 (43.13% ± 2.82) and -13 (54.54% ± 2.12) [72,73,74]. Additionally, in studies conducted by Osei Akoto et al., ethanol and hexane extracts showed anti-inflammatory properties. The anti-inflammatory activity levels of ethanol and hexane extracts were significantly higher than that of aspirin (comparator) at test concentrations. Standard aspirin and ethanol and hexane extracts showed concentration-dependent anti-inflammatory efficacy at test concentrations of 500–1000 μg/mL [75]. The presence of glycosides, phenols, terpenoids, flavonoids and steroids in *Ocimum basilicum* explains the anti-inflammatory activity of the plant. Flavonoids not only inhibit enzymes, but also inflammatory mediators, such as adhesion molecules and C-reactive protein.

Acute and chronic in vivo testing confirmed the anti-inflammatory effects of *Ocimum basilicum* essential oil (EOOB) and the main component estragole in models of paw edema, vascular permeability, granulomatous inflammation and peritonitis. The involvement of the histamine and arachidonic acid pathways has been used to study the mechanism of anti-inflammatory action. Paw edema caused by carrageenan and dextran was significantly reduced by treatment with EOBB (100 and 50 mg/kg) and estragole (60 and 30 mg/kg). Doses of EOBB (50 mg/kg) and estragole (30 mg/kg) were most effective in preventing arachidonic acid and histamine-induced paw edema, inhibition of leukocyte emigration into the peritoneal fluid, and vascular permeability. These doses were able to reduce chronic inflammation. EOOB and estragole have demonstrated anti-inflammatory efficacy, but essential oil has been shown to be more effective in acute and chronic anti-inflammatory effects [76,77].

Okoye-Festus et al., in their studies, evaluated the topical anti-inflammatory effects of volatile components extracted from fresh leaves of *Ocimum basilicum*. Fresh plant leaves were subjected to hydrodistillation to obtain volatile oils (OBV). Additionally, n-hexane (OBHE) was used to extract fresh leaves. OBV and OBHE were tested for anti-inflammatory effects using xylene-induced ear edema as an inflammation model. At a dose of 50 μg/ear, OBV and OBHE had significant (*p* < 0.05) local anti-inflammatory effects, with suppression of edema by 62.7 and 80%, respectively. The effects which were observed were comparable (*p* < 0.05) to that of hydrocortisone, at 100 μg/ear (% swelling inhibition 54.8) [78].

An in vitro study of the anti-inflammatory effect of *Ocimum basilicum* in obese patients also showed good results. Fresh leaves of *Ocimum basilicum* were lyophilized, processed, and extracted with 80% methanol. Following a 24 h culture period of 3T3-L1 adipocytes with *Ocimum basilicum* extracts at final concentrations of 5 or 25 μg/mL, RAW264.7 macrophages were plated on these adipocytes and co-cultured for 12 h. The effects of *Ocimum basilicum* extracts on the expression of inflammatory cytokines were determined using real-time PCR or western blotting. Extracts from *Ocimum basilicum* decreased the mRNA expression of inflammatory cytokines, such as TNF-α (Tnf), CCL2 (Ccl2), IL-1β (Il1b) and IL-6 (Il6), that were produced by co-cultivation. Furthermore, the mRNA expression of NF-κB (Nfκb1), a transcription factor for inflammatory cytokines, was decreased by *Ocimum basilicum* extracts. In a study of co-stimulatory inflammatory signaling CD137 (Tnfrsf9)/CD137L, a member of the TNF superfamily, *Ocimum basilicum* extracts inhibited co-culture-induced Tnfrsf9 expression. This demonstrates that the main mechanism by which *Ocimum basilicum* extracts exert anti-inflammatory effects on adipocytes is by suppressing the inflammatory signaling pathway by reducing Tnfrsf9 gene expression. Treatment with *Ocimum basilicum* extracts also resulted in a decrease in Nfκb1 gene expression, which also shows that NF-κB signaling is inhibited by suppressing the expression of the upstream regulator CD137 [79].

A study of the anti-inflammatory effect of *Ocimum basilicum* essential oil in combination with β-cyclodextrin (OBEO/b-CD) in mice showed good results. Complexation with b-cyclodextrin (b-CD) was carried out using various methods and analyzed by thermogravimetry (TG), differential scanning calorimetry (DSC) and scanning electron microscopy (SEM). Anti-inflammatory activity was assessed in mouse models of paw edema induced by dextran, carrageenan, arachidonic acid (AA) and histamine; peritonitis and vascular permeability caused by carrageenan and granuloma due to cotton-block insertion. DSC, TG and SEM analyses showed that OBEO successfully complexed with b-CD. Oral administration of OEOB/b-CD, by reducing vascular permeability in vivo, prevented the formation of paw edema, inhibited the formation of granulomas in mice, and inhibited the recruitment of leukocytes into the peritoneal cavity. The results show that b-CD conjugation improves the anti-inflammatory effects of OBEO in mouse models of acute and chronic inflammation, indicating that this complex can be used in the development of anti-inflammatory drugs [80].

## 8. Wound Healing Effect

A study evaluating the effectiveness of topical application of *Ocimum basilicum* emulgel for wound healing in animal models showed wound healing activity. The produced formulations (OB-emulgel) were assessed for appearance, rheological behavior, patch/sensitivity, in vitro drug release, FT-IR analysis, stability and spreadability. Macroscopic and histological results were used to assess the in vivo wound healing efficacy in rabbit wounds and compare it with the commercially available Quench^®^ silver sulfadiazine cream. Results from experimental and standard animal models showed a significant (*p* < 0.05) increase in wound contraction velocity. The percentage of wound reduction in the standard treatment group was higher throughout the entire treatment period, and amounted to 51 ± 0%, 69.75 ± 4.29%, 91 ± 0% and 99 ± 0.24%, but on the 8th day, this figure in the extract treated group was higher than that in the standard treatment group, which was 69.75 ± 4.29%. But this increase was not significant (*p* > 0.05). The rate of wound contraction between the group receiving the extract and the group receiving standard treatment was not significant (*p* > 0.05). The high rate of wound healing in experimental animal models is due to three factors: the presence of phytochemicals, such as estragole and linalool, which have the ability to heal wounds and at the same time have an anti-inflammatory effect, increased epithelialization, and increased angiogenesis [81].

In another study, a new gel formulation was prepared using a combined extract of *Ocimum basilicum* and Trifolium pratense (EOT), and was subsequently tested in vitro (using a scratch test) and in vivo (an animal model) to demonstrate its beneficial effects on pathological skin. In vitro experiments revealed that EOT at a dosage of 50 μg/mL completely restored the dermal fibroblast monolayer. In vivo tests using the EOT-based hydrogel composition demonstrated improved wound closing time and full healing after 13 days of therapy. This effect is explained by the synergistic action of the combined compounds obtained from the plant mixture. The positive in vivo findings are attributed not just to the synergy of the two extracts employed in the EOT gel recipe, but also to the body’s immunological response. The use of EOT affects both the migration and the proliferation of fibroblasts, as the immune process and growth factor levels are involved. In addition, a clinical case of psoriasis vulgaris was presented in which one week of treatment led to a significant improvement in the patient’s health [82].

The wound-healing properties of an aqueous extract of *Ocimum basilicum* were studied in 120 male Sprague–Dawley rats. After creating skin wounds, the animals were randomly divided into four groups: untreated control, treated with Eucerin ointment, treated with 3% tetracycline ointment, and treated with 3% ointment with aqueous extract of *Ocimum basilicum*. Skin sections of all thicknesses were prepared 10, 20 and 30 days after wound creation for biochemical and histopathological analysis of skin wound healing trends. *Ocimum basilicum* aqueous extract ointment significantly (*p* < 0.05) decreased the levels of wound area, total cell count, neutrophils and lymphocytes, and increased the levels of wound contracture, hexuronic acid, hydroxyproline, fibrocytes and hexosamine, compared with the control group [83]. Also, to evaluate the wound-healing activity on an excised wound model, an aqueous–alcoholic extract of the aerial part of *Ocimum basilicum* was used. An ointment from an aqueous–alcoholic extract of the aerial part was applied to a model of an excised wound until complete epithelialization, and assessed by various parameters, such as measuring the area of the wound, percentage of wound reduction, and epithelization period. As the results showed, there were significant decreases (*p* < 0.01) in wound area (mm^2^) in the experimental groups compared to the control, which was measured at 0, 4, 8, 12 and 16 days after wounding. The rate of epithelization was higher in animals of the experimental groups, compared to the control group. According to the authors, the aqueous–alcoholic extract of the aerial part of *Ocimum basilicum* contains phytocomponents responsible for reducing wounds and increasing the rate of epithelization. The hydroalcoholic extract of the aerial part of *Ocimum basilicum* contains steroids, flavonoids, tannins and proteins. Flavonoids improve vascularization and avert cell necrosis, which both lower lipid peroxidation. Due mostly to their astringent and antibacterial qualities, tannins and flavonoids accelerate the healing processes of wounds [84].

## 9. Antiviral Activity

*Ocimum basilicum* extracts and purified components were utilized to find potential mechanisms of antiviral action against RNA viruses (Enterovirus 71 (EV71) and Coxsackievirus B1 (CVB1)) and DNA viruses (hepatitis B virus, adenoviruses (ADV) and herpes viruses (HSV)). The antiviral activity of *Ocimum basilicum* was demonstrated using a range of crude ethanolic extracts, an aqueous solution, and specific purified components, such as linalool, apigenin and ursolic acid. Crude extracts obtained from *Ocimum basilicum* exhibited anti-adenoviral activity, and the purified components apigenin, ursolic acid, and linalool had strong antiviral activity against all types tested. Ursolic acid had the most effectiveness against HSV-1 (EC_50_ = 6.6 mg/L; selectivity index (SI) = 15.2), ADV-8 (EC_50_ = 4.2 mg/L; SI = 23.8), CVB1 (EC_50_ = 0.4 mg/L; SI = 251.3) and EV71 (EC_50_ = 0.5 mg/L; SI = 201); while apigenin demonstrated the greatest activity against HSV-2 (EC_50_ = 9.7 mg/L; SI = 6.2) and ADV-3 (EC_50_ = 11.1 mg/L; SI = 5.4); and linalool showed the greatest activity against hepatitis B e-antigen (EC_50_ = 12.8 mg/L; SI = 1.3), hepatitis B surface antigen (EC_50_ = 7.1 mg/L; SI = 2.3) and AVD-II (EC_50_ = 16.9 mg/L; SI = 10.5). An aqueous extract of *Ocimum basilicum* showed broad, albeit modest, anti-adenoviral activity. No activity was observed for β-caryophyllene, carvone, farnesol, cineole, fenchone, geraniol, α-thujone, or β-myrcene. Furthermore, ursolic acid has been shown to inhibit CVB1 and EV71 during both the infection and replication processes [85].

Studies analyzing the inhibition mechanism of three polyphenolic compounds of *Ocimum basilicum*, namely, apigenin-7-glucuronide, dihydrokaempferol-3-glucoside and esculetin, against the SARS-CoV-2 virus have shown good results. Ritonavir was used as a positive control because it is a protease inhibitor that is used to treat HIV and has antiviral activity against SARS-CoV-2 viruses. The three inhibitor compounds were compared using ritonavir’s binding affinity as an indication. Apigenin-7-glucuronide and dihydrokaempferol-3-glucoside showed better efficacy than did esculetin. They have binding affinities that are not significantly different from ritonavir. The binding affinity of apigenin-7-glucuronide was −8.77 kcal/mol, dihydrokaempferol-3-glucoside—−8.96 kcal/mol and esculetin—−5.79 kcal/mol. The values of the inhibition constants were 375.81 nM, 270.09 nM and 57.11 μM, respectively. Apigenin-7-glucuronide and dihydrokaempferol-3-glucoside interacted more with the amino acids of the Mpro enzyme through hydrogen bonds than esculetin. This was affected by the hydroxyl group which was present in the molecule. Dihydrokaempferol-3-glucoside and apigenin-7-glucuronide have more hydroxyl groups than esculetin, which resulted in stronger antiviral activity. The antiviral mechanism was provided by the quantity of hydroxyl groups associated with a benzene ring. When analyzed by ADMET, these three compounds were consistent with the predicted pharmacokinetic parameters. Moreover, in the analysis of drug similarity, the compounds apigenin-7-glucuronide and dihydrokaempferol-3-glucoside had one violation, while esculetin had no violations [86].

The antiviral activity of eugenol and eugenol epoxide isolated from *Ocimum basilicum*, assessed using a set of HIV-1 p24 antigens, showed that eugenol and eugenol epoxide inhibited viral replication by more than 90%, at concentrations of 500 μg/mL. These two compounds’ levels of antiviral activity were investigated further at various doses of 250, 100 and 50 μg/mL. The effective doses for reducing viral titers by 50% (EC_50_) for eugenol and eugenol epoxide were 350 and 80 μg/mL, respectively, and the selectivity indices (SI) was 4.2 and 21.25, respectively. The time-addition experiment was performed by measuring viral RNA yield in infected culture supernatants using real-time PCR analysis. Eugenol and eugenol epoxide were added before (−12 and −6 h), during (0 h), and after (6 and 12 h) virus absorption into the culture medium, until the end of the experiment. There was a marked reduction in the amount of HIV-1 DNA in the treated cultures, compared to control viruses. The strongest proliferations of HIV-1 in cells treated with eugenol and eugenol epoxide were observed when extracts were added before and during the initial stages of infection, respectively. The amount of viral DNA in eugenol-treated cells was 104–105 times lower than in untreated controls 12 and 6 h before infection. In comparison to untreated control cells, the amount of DNA in eugenol-epoxide-treated cells was 104 times less during infection [87].

A study of the effect of using an alcoholic extract of *Ocimum basilicum* on the antiviral activity against the Newcastle disease virus of a monolayer culture of chicken embryo fibroblasts in vitro before and after inoculative treatment of fibroblast cells with plant extracts showed that the leaves of *Ocimum basilicum* have antiviral activity against the Newcastle disease virus in doses of 500 and 250 µg/mL. However, the virus reduction titer was found to range from 10–6 to 10–1 when using *Ocimum basilicum* extract at a concentration of 500 μg/mL, compared to 10–7 when using the viral control [88].

The antiviral activity of hydrodistilled essential oils of *Ocimum basilicum* (herb) was determined using cytopathicity assay (CPE) against herpes simplex virus 1 (HSV-1). Incubation of virus-infected cells with essential oils increased the viability of these cells, compared to untreated virus-infected cells (control). Antiviral activity increased with increasing concentrations of essential oils. The addition of 200, 500 and 1000 μg/mL of *Ocimum basilicum* essential oil increased the percentages of antiviral activity to 37.66, 40.20 and 80.75%, respectively. According to the authors, the antiviral properties of essential oil are explained by the active action of linalool, the main chemical component of essential oil [89,90].

The antiviral activity of *Ocimum basilicum* essential oil against BVDV was investigated by KubiçaI et al. Bovine viral diarrhea virus (BVDV) has been taken as a model for antiviral studies of hepatitis C virus (HCV). Three monoterpenes were the primary constituents of the *Ocimum basilicum* essential oil employed in the study: linalool, camphor (12.80%), and 1,8-cineole (23.61%). Antiviral activity was tested using a plaque reduction assay. Based on the quantity of plaques in the viral control, the percentage of plaque inhibition for each component was calculated. Camphor and 1,8-cineole showed the highest antiviral activity (camphor IC = 13.88 and 1,8-cineole IC = 9.05), with a virucidal effect. The higher levels of activity of monoterpenes in the virucidal test indicates that these compounds act directly on the viral particle [91].

## 10. Conclusions

The medicinal potential of *Ocimum basilicum* is varied and constantly evolving. Most of the pharmacological properties of *Ocimum basilicum* have been demonstrated, both in vivo and in vitro. Its broad medical effectiveness has been confirmed by several clinical trials. However, more similar studies are needed to determine and confirm the ethnopharmacological profile of *Ocimum basilicum*. Therefore, more studies in clinical disease models are needed to evaluate and confirm the effectiveness of plants in treating various diseases. It is important to note that, unlike other drugs used to treat the disease, the healing effects of herbs and their extracts do not cause side effects. But it must be taken into account that, although the active ingredients come from natural sources, high dosages can cause harmful side effects. Large-scale synthesis, chemical characterization, therapeutic evaluation, and toxicity studies should be major areas of future research. In addition, certain explanatory mechanistic studies need to be conducted to determine the mechanism of action of this medicinal herb. This will confirm traditional knowledge about this revered medicinal herb.

## Figures and Tables

**Table 1:** Major biological active compounds of *Ocimum basilicum*.

Extracts	Plant Part	Method	Biological Active Compounds	Pharmacology Activity	Country	Ref.
Essential oil	Leaves, seed, root	GC/MS	Menthone (33.1%), oxygenated monoterpenoids (77.8%), estragole (21.5%), oxygenated monoterpenes (75.3%), isoneomenthol (7.5%), transcaryophyllene (2.2%), menthol (6.1%), limonene (1.5%), pulegone (3.7%), sesquiterpene hydrocarbons (8.8%), trans-β-farnesene (1.1%), germacrene D (1.4%), α-amorphene (1.1%), menthyl acetate (5.6%), α-cadinol (2.9%), methyl eugenol (1%), sesquiterpenoids (12.8%).	Antioxidant, Antimicrobial, Ani-Inflammatory	Iran	[32]
Essential oil	Leaves, seed	GC/MS	Bolloso Napoletano: linalool (47.75%), 1,8-cineole (10.23%), methyl chavicol (20.21%); Foglie di Lattuga: linalool (48.65%), 1,8-cineole (12.59%), methyl chavicol (18.55%). Thai Siam: linalool (36.60%), methyl chavicol (7.50%), (*E*)-methyl cinnamate (21.90%).	Antioxidant, Anti-Inflammatory, Antiviral	Poland	[33]
Etanolic, Metanolic	Stem, seed	HPLC, GC/MS	1,6-octadiene-3-ol, 3,7-dimethyl (29.49%), eucalyptol (3.31%), cis-alpha-bisabolene (1.92%), trans-alpha-bergamotene (5.32%), beta-myrcene (1.11%), levomenthol (1.81%).	Antimicrobial, Antioxidant	South Africa	[34]
Etanolic, Metanolic	Leaves, seed, root	HPLC, GC/MS	1,8-cineole (10.56%), linalool 48.4%, methyl chavicol 14.3%, α-bergamotene 27%, oxygen monoterpenes (57.42%), β-bisabolol 4.1%, methyl eugenol (10.09%), stragol (55.95%), sesquiterpene hydrocarbons (6.9%).	Antioxidant, Anti-Inflammatory, Antifungal	Egypt	[35]
Essential oil	Leaves	GC/MS	Linalyl acetate (19.1%), linalool (52.1%). Aliphatic compounds (9980–17,929 nanograms per gram fresh weight), including (*E*)-2-hexenal: 1519–1991, (*Z*)-3-hexenal (4991–10731), (*E*)-2-hexen-1-ol: 75–144, (*Z*)-3-hexen-1-ol: 1436–2219, n-hexanol: 73 –175, 1-octen-3-ol: 1610–2689, (*Z*)-3-hexenyl acetate: 54–99; eugenol (66,142–131,926). α-pinene: 875–1198, camphene: 153–295, β-pinene: 1780–2771, 2-carene: 42–142, myrcene: 2770–3030, limonene: 712–870, 1,8-cineole: 26,640–52,799, 3-carene: 41–48, linalool: 42,726–65,033, bornyl acetate: 332–1163, camphor: 164–463, tepinen-4-ol: 185–364, eugenol: 945 –1948, α-terpineol: 159–310, α-bergamotene: 202–406 and (*E*,*E*)-α farnesene: 32–65), α-humulene: 141–538, caryophyllene: 641–1432.	Wound Healing, Antiviral, Antimicrobial	Algeria	[36,37]
Essential oil	Leaves	HPLC, GC/MS	Linalool and 1,8-cineole.	Antimicrobial and Antioxidant	Serbia	[38]
Essential oil	Leaves, stem	HPLC, GC/MS	Limonene (30.9%), p-cymene (2.6%), linalool (18.9%), thymol (6.5%), B-phellandrene (15.3%), O-cardinol (2.6%).	Antimicrobial and Antioxidant	Cameroon	[39]
Etanolic, n-hexane	Leaves, stem	TLC, HPLC	Estragole (>35.71%), trans-α-bergamotene (>0.83%), (*E*)-β-ocimene (>1.47%), eucalyptol (>0.25%), τ-cadinol (>0.41%).	Antimicrobial and Antioxidant	Malaysia	[40]
Essential oil	Leaves	FT-IR, GC/MS	Eugenol (61.76%), [2-methyl-4-(1))-propyl)phenoxy]silane (2.01%), 2,3-dihydroxypropyl elaidate (5.10%), isopropyl palpitate (11.36%), 2-methoxy-4-(1-propyl)phenol (2.65%), α-cubene (3.85%), vanillin (1.27%), 1-methyl-3-(1-methyl)benzene (1.73%), 1,4-diethylbenzene (1.03%), hexadecanoic acid methyl ester (2.51%).	Wound Healing	Bangladesh	[41]
Essential oil	Leaves	HPLC	Methyleugenol (15.5%), patchoulan (6.7%), 2-phenyl-1-hexanol (14.0%), o-nitrocumene (14.0%), 2-methyl- 3,5-dodecadiine (14.0%), 1-(4,5-dimethyl-2-nitrophenyl)-1*H*-tetraazole (14.0%).	Antimicrobial and Antioxidant	Nigeria	[42]

**Table 2:** Antimicrobial activity of *Ocimum basilicum* extracts.

Tested Microorganism	Ethanolic Extract	Methanolic Extract	Aqueous Extract	Acetone Extract	Linalool	Ref.
Diameter of inhibition zone (mm)						
*S. aureus*	20.4 ± 1.0	26.9 ± 1.2	24.1 ± 1.2	21.2 ± 1.2	26.1 ± 1.1	[44,45,46]
*P. multocida*	24.4 ± 1.1	25.3 ± 1.1	23.2 ± 1.4	22.2 ± 1.3	24.0 ± 1.0	[43]
*B. subtilis*	13.2 ± 0.8	19.5 ± 1.1	13.5 ± 0.8	11.4 ± 0.6	16.2 ± 1.0	[44]
*E. coli*	13.6 ± 0.8	22.3 ± 1.0	18.4 ± 1.0	16.1 ± 1.0	18.0 ± 0.9	[44]
*M. mucedo*	19.4 ± 1.1	21.4 ± 1.0	17.7 ± 1.3	15.2 ± 0.7	11.7 ± 0.7	[43]
*A. niger*	21.6 ± 1.2	23.3 ± 0.8	20.4 ± 1.2	18.4 ± 1.2	18.7 ± 0.7	[43]
*F. solani*	13.6 ± 0.8	11.2 ± 0.6	9.7 ± 0.6	11.1 ± 0.9	9.7 ± 0.6	[43]
*R. solani*	17.2 ± 1.0	17.6 ± 1.0	16.6 ± 1.0	14.3 ± 1.1	13.6 ± 0.8	[43]
*B. theobromae*	13.5 ± 0.8	17.3 ± 0.8	14.3 ± 0.8	12.3 ± 0.7	10.3 ± 0.6	[43]
Minimum inhibitory concentration (mg/mL)						
*S. aureus*	1.2 ± 0.0	0.8 ± 0.0	0.8 ± 0.0	1.4 ± 0.0	0.3 ± 0.0	[44,45,46]
*P. multocida*	1.5 ± 0.0	0.9 ± 0.0	1.1 ± 0.0	1.3 ± 0.0	0.4 ± 0.0	[43]
*B. subtilis*	0.06 ± 0.1	0.03 ± 0.1	2.0 ± 0.1	2.6 ± 0.1	0.9 ± 0.0	[44]
*E. coli*	2.2 ± 0.1	4.5 ± 0.2	2.7 ± 0.1	3.2 ± 0.2	1.0 ± 0.1	[44]
*M. mucedo*	2.0 ± 0.1	1.7 ± 0.1	2.3 ± 0.1	1.9 ± 0.1	0.9 ± 0.0	[43]
*A. niger*	3.0 ± 0.2	5.0 ± 0.3	2.9 ± 0.2	4.3 ± 0.2	1.5 ± 0.1	[43]
*F. solani*	2.7 ± 0.1	4.9 ± 0.2	3.2 ± 0.2	3.6 ± 0.2	1.6 ± 0.1	[43]
*R. solani*	2.3 ± 0.1	4.6 ± 0.2	2.9 ± 0.2	4.1 ± 0.2	1.1 ± 0.0	[43]
*B. theobromae*	3.8 ± 0.2	5.1 ± 0.3	4.6 ± 0.2	4.9 ± 0.3	1.9 ± 0.1	[43]

## Data Availability

Not applicable.
